# Hepatitis B Therapeutic Vaccine: A Patent Review

**DOI:** 10.3390/ph15121542

**Published:** 2022-12-12

**Authors:** Shuaibu Abdullahi Hudu, Abdulgafar Olayiwola Jimoh, Kasimu Ghandi Ibrahim, Ahmed Subeh Alshrari

**Affiliations:** 1 Department of Basic Medical and Dental Sciences, Faculty of Dentistry, Zarqa University, Zarqa 13110, Jordan; 2 Department of Pharmacology and Therapeutics, Faculty of Basic Clinical Sciences, College of Health Sciences, Usmanu Danfodiyo University, Sokoto 840001, Nigeria; 3 Department of Basic Health Sciences, Faculty of Pharmacy, Northern Border University, Rafha 91911, Saudi Arabia

**Keywords:** therapeutic, vaccine, patent review, hepatitis B infection, clinical trial

## Abstract

Viral hepatitis has long been underrated as a danger to global health. The UN only recently called for worldwide action to tackle viral hepatitis and lessen the disease burden in its “2030 Agenda for Sustainable Development”. Hepatitis B virus (HBV), which causes liver cirrhosis and malignancy, is a main cause of death globally. This review analyses innovative HBV therapeutic vaccine candidates for which a patent was filed between January 2010 and March 2022 and presents future improvement techniques for vaccine efficacy. Although there is a preventative vaccine for HBV infection, over 3% of people worldwide have the disease on a long-term basis and can no longer benefit from it. Most people will have chronic HBV infection for the rest of their lives once it has been diagnosed. Moreover, only a small percentage of treated patients experience a functional cure with persistent hepatitis B surface antigen reduction. A significant proportion of deaths are caused by liver cirrhosis and hepatocellular cancer, which are both caused by chronic hepatitis B infection. Hence, there is an urgent need for novel medications due to the inadequacies of the current therapies.

## 1. Introduction

Hepatitis B virus (HBV) is one of the tiniest enveloped viruses, with an estimated bread of about 42 nm and an estimated length of 3200 nucleotides, and with partially double-stranded round deoxyribose nucleic acid (DNA). The viral molecule (virion) has an icosahedral nucleocapsid, external lipid envelope, genomic DNA and DNA polymerase which has reverse transcriptase action [1]. Hepatitis B infection has been classified into various genotypes dependent on differences in the nucleotide sequence of more than 8% [2]. Eight genotypes designated A through H have been identified in this regard [3,4]. In addition, genotype J has been identified in Japan [5] and genotype I in Vietnam, both of which are unknown and likely recombinant [6]. Among the most common viral infections in the entire world is HBV infection. Over two billion people globally are infected, with about 240 million having chronic hepatitis B infection. It has been estimated that 3.9% of men and 3.5% of females are chronically infected with HBV globally [7]. The immune system recognizes and attacks HBV by activating CD4+ and CD8+ cells that recognize diverse HBV-derived peptides on the surface of hepatocytes and inflicting liver damage as a result of an immune response. Host immune interaction with HBV plays an important role in the pathogenesis of the disease [8,9,10].

Active hepatitis B infection occurs within the first six months, with almost 90% of adults developing a comprehensive, multi-specific cellular immunity that helps in clearing the virus, eventually leading to the development of anti-HBs [11,12,13]. Persistence of the virus beyond six months of infection is considered a chronic infection and around 1% of those with acute icteric infection may end up developing fulminant hepatitis B infection. Acute HBV infection has no specific treatment [14,15]; as such, its management is purely supportive while chronic hepatitis B treatment is aimed at sustained suppression of viral replication to reduce the rate of developing cirrhosis, decompensation, and risk of hepatocellular carcinoma [16,17,18]. There are two fundamental choices for treating chronic HBV infection, nucleos(t)ide analogs (NAs) or alpha-interferons (IFNs), which are also used in personalized medicine [19,20,21,22]. By inhibiting DNA polymerase, NAs provide an immediate antiviral effect against HBV when taken orally [23,24]. Tenofovir disoproxil fumarate (TDF), telbivudine (TBV), entecavir (ETV), lamivudine (LAM), adefovir (ADV), and tenofovir alafenamide (TA) are the six different approved NAs for hepatitis B treatment. Telbivudine, adefovir, and lamivudine have low resistance barriers and their usage is no longer advised, while tenofovir and entecavir are highly resistant to mutation, and are therefore recommended [23,25,26,27].

There are two forms of HBV vaccinations: recombinant yeast-derived vaccines, introduced in 1986, and plasma-derived vaccines, which have been in use since 1982 [28,29]. An efficient method of avoiding HBV infection is the recombinant HBsAg protein found in the hepatitis B vaccine [30]. The HBsAg protein is primarily targeted by the anti-HBs antibody found in HBIg and antibodies activated by active hepatitis B vaccination [31]. However, it has been discovered that mutations that occur near the determinant change how antigenic the hepatitis B surface protein is, which can then prevent anti-HBs antibodies from neutralizing HBV [32,33]. Since the HBV surface mutation is becoming a global issue and the mutants rarely respond to the currently available vaccines, it is necessary to develop newer vaccines. Several vaccines are currently being tested, such as HEPLISAV-B, which is the first new HBV vaccine to be approved in more than 25 years and is only given to adults in two doses [34].

HepB-CpG has been demonstrated to be as immunogenic as the Engerix-B vaccine; nonetheless, its long-term safety and the persistence of immune memory cells have not been established [35]. In a phase 1 clinical trial, Zoulim and Fournier [36] examined the safety, immunogenicity, and tolerability of the TG1050 vaccine for one patient with chronic HBV infection. The TG1050 vaccine is adenovirus 5-based, expressing HBV polymerase and the surface antigen domain. Similarly, Bian and Zhang [37] explored whether preS1-polypeptide inoculation could serve as a possible therapeutic vaccine for one patient infected with HBV. The clinical trial results showed that the preS1 area of L-HBsAg presents solid immunogenicity for both B-cell and T-cell reactions. The core tenet of a patent examination is based on the idea that referencing a patent entails including information components from the cited patent, allowing the development cycle to be proved by an organization [38]. In this way, we can utilize a patent reference network to provide an appraisal of patents granted for hepatitis B viral vaccines, targeting the period from 2010 to 2020, and advise pharmaceutical companies, policymakers, and researchers interested in HBV vaccine development.

## 2. Patent Search

The search for patents was conducted on 30 September 2022. In the Espacenet database, a search for “therapeutic vaccine” produced 87210 hits, while a search for “therapeutic vaccination with HBV” produced 8467 items. A similar comparison was made between searches for “therapeutic vaccination” and “HBV” in the Patentscope database, which produced 4823 hits for the former and 62 finds for the latter. For the combo search, the Sci-finder search returned 256 results. The choice of databases for the search was based on availability, accessibility, and convenience. All of the therapeutic HBV vaccines were built on various platforms, including protein subunits, viral vectors, DNA, attenuated viruses, RNA, viral vectors, virus-like particles, and live attenuated viruses. Some of the vaccines are currently in preclinical development, while others are currently in clinical trials. The names of the principal developers and the platform technology were used to categorize the resulting patents and patent applications. Patent families were divided and the redundant references were eliminated. We also looked through the websites of the companies that produce vaccines to learn about any assigned patents.

## 3. Analysis

Currently, no curative treatment is available for patients with chronic hepatitis B infection [18]. Many organizations have halted research of promising products even after exciting Phase I/II results because they were unable to meet particular clinical endpoints for further trials. Therapeutic vaccines have been improved by the introduction of new antigens, various administration methods, adjustments to vaccination schedules, the addition of powerful adjuvants, and dosage adjustment of the antigen [39,40,41].

The fact that these clinical studies were carried out on people with severe and long-lasting viral suppression was one of the factors contributing to their failure [42,43,44]. It has been found that a decrease in viral load occurs frequently before T-cells respond specifically to anti-HBs in patients receiving HBeAg and naturally resolve the infection [45,46]. This strengthens the argument for therapeutic vaccine needs. To improve the reactivity of T-cells specific to hepatitis B that have turned non-responsive during chronic hepatitis B infection, nucleotide analog antiviral medicine must be utilized to reduce the HBV burden [47,48]. However, the immune system is impacted by fluctuations in viral load. Early therapeutic vaccination studies were conducted on prophylactic recombinant vaccines including HBV envelope proteins expressing HBsAg. Considering that the two main outcomes of any anti-HBV therapy are the elimination of HBsAg and the generation of protective anti-HBs antibodies, the initial decision seemed fair. Receiving the recommended vaccine considerably enhanced the proportion of HBeAg seroconversion according to pilot clinical studies [49,50]. Several strategies were employed by researchers in developing HBV therapeutic vaccines using one or more hepatitis B viral proteins in different platforms with specific interventions, as shown in Table 1.

To date, no approved vaccine has been shown to have therapeutic potential to heal hepatitis B virus-infected people who are chronically unwell [39,40,51]. In a preclinical Phase I experiment involving 15 healthy volunteers, a possible therapeutic vaccine (Heptavax-B) contained a recombinant chimeric molecule produced in insect cells that contains a hepatitis B viral antigen and a portion of a murine monoclonal antibody that targets dendritic cells, which are important in antigen presentation and the beginning of an immune response that includes cellular and humoral reactions [52]. The clearance of HBsAg and production of anti-HBs was seen in only a few patients; hence, there is a need for a therapeutic vaccine with better efficacy. To increase therapeutic vaccine effectiveness, both vaccine composition and administration techniques have been proposed.

A Phase IIB clinical study that was double-blinded, placebo-controlled, and which enrolled 242 individuals with chronic hepatitis B infection was conducted [53]. Antigen-antibody complexes with alum as the adjuvant were administered intravenously to these individuals. Large levels of IL-12 are secreted by dendritic cells (DCs) when treated with antigen-antibody complex. They are believed to activate CD8 T cells in vivo and upregulate functional markers in vitro. Twenty-four weeks after the final injections, a significant virological effect was seen. However, the therapeutic effects and the immune response were not analyzed in the study [53]. Numerous researchers had evaluated possible therapeutic vaccines for long-term HBV treatment over the past 20 years and many patents were filed (Table 2). Some regimens have demonstrated the ability to reduce viral load and enhance specific immune responses to HBV; however, none have been effective in causing a complete remission to date.

### Future Perspective

The analysis of the possibility and limits of therapeutic vaccines suggests that they are both safe and endowed with antiviral and liver-protective potential. The majority of the therapeutic vaccines also show little potential in reducing hepatitis B viral load, hence the need to come up with an effective and safe therapeutic vaccine that is of great benefit to chronic hepatitis B patients. The following should therefore be considered in designing a suitable hepatitis B therapeutic vaccine: (a) the nature of the antigen, because several data points suggest that HBsAg-based vaccination therapy alone may not be the best form of vaccine therapy since HBsAg-based immunity is not likely to affect internalized HBV DNA and cccDNA, (b) more emphasis should be placed on the use of HBcAg or other HBV-related antigens as potential antigens for vaccination therapy, notwithstanding the usage of HBsAg-based vaccine therapy, (c) studies on vaccination therapy should be adequately planned in terms of the dosage and length of the course of treatment, studies should be designed to offer information on safety and effectiveness for both short- and long-term treatment, and (d) when vaccination therapy shows antiviral and liver protection, the mechanisms underlying such activities should be adequately investigated.

## 4. Conclusions

Numerous approaches have been suggested to boost the effectiveness of therapeutic immunization against chronic HBV. Even though most of the hepatitis B therapeutic vaccines are specific and sensitive in chronic hepatitis B patients, they still cannot induce an HBV T cell-specific immune response that is capable of providing a cure. Therefore, all possible approaches to improving therapeutic vaccination focus on enhancing vaccine-induced innate immunity and eliminating the HBV-specific T-cell inefficiency brought on by chronic infection.

## 5. Expert Therapeutic Opinion

HBV vaccination safety has been acknowledged worldwide as a standard practice. The goal of such vaccination is to induce a host immune response, thereby averting the replication of HBV in the host. There are a few immunological and clinical variables that decide the clinical adequacy and safety of the HBV antibody. At present, the approved recombinant hepatitis B vaccines are immunogenic, effective in over 90% of vaccinees, and by and large very well endured, as demonstrated by over twenty years of a successful vaccination program. In this way, the most attractive improvement is to stretch out its efficacy to 100% of the vaccinees. To this end, planned endeavors with a novel vector for the effective articulation of HBV recombinant antibodies have shown potential. A critical number of patent revelations have described efficacy and immunogenicity improvements by utilizing novel vector frameworks to invigorate humoral and cellular immune responses. A significant expansion in immunogenicity, particularly the expansion of a huge cell-interceded reaction, would likewise assist in decreasing the number of doses expected to achieve a level of protective immunity. Although the products may have somewhat different conformations, such as modified lipid components, we do not agree that the manufacture of a similar HBsAg antigen using enhanced cleaning procedures carries many guarantees for boosting the immunogenicity of the existing vaccines. Another procedure has been to purify various antigens, for example, the nucleocapsid comprised of the HBV core antigen or lipoprotein particles containing preS proteins, which have indicated promising immunogenicity.

Most of the recently disclosed novel vector expression frameworks and adjuvants, as well as certain peptides, transgenic plants, nucleotides, the PreS, L, and s regions of HBsAg, and a mix of mutants and wild-type HBV in a bivalent recombinant vaccine have shown little or no benefit in therapeutic vaccine development. Hansenula is the leading HBsAg expression system and is also an adjuvant, with the unmistakable preferred position that it has been administered to a vast number of human volunteers for different antigens. A United States patent with publication number US10653772B2 described a hepatitis B treatment antibody based on inactivated, entire recombinant Hansenula cells expressing HBsAg, and the immunization utilizes enhanced, inactivated, completely recombinant Hansenula polymorpha cells as an adjuvant. The advancement in DNA recombinant technology, gene editing, reverse genetics technology, and the advancement of recombinant live vector vaccines is drawing increasing attention. The recombinant live vector antibody is a live vaccine expressed by embedding an exogenous protective antigen into a live vector genome by utilizing a genetic engineering innovation, and mostly involves recombinant viral and bacterial vector live vaccines. A China patent with dissemination number CN111363728A reports the use of recombinant influenza A virus as a vector that incorporates the genome of HBV into the influenza genome. This is achieved by using a reverse genetics innovation to ensure that the recombinant flu virus can be steadily passaged in host cells or chick embryos. This can then be utilized for creating a hepatitis B vaccine and related medicaments and conveying hepatitis B-related proteins by using the chick cells or embryos as bioreactors.

The area of therapeutic vaccine efficacy for the treatment of an infected patient with no symptoms is neglected. However, vaccination derives both its therapeutic and prophylactic effectiveness from the curing of existing infection and the prevention of re-infection. Therefore, consideration of vaccine efficacy in asymptomatic patients would have an immensely positive effect on an enormous number of HBV asymptomatic carriers on the planet. A China patent disclosure identifies a live HBV therapeutic vaccine prepared by expressing the outer membrane protein complex of the hepatitis B surface antigen. The mixture consists of the epitope protein on an *Escherichia coli* cell in which the HBsAg antigenic determinant peptide is embedded into the fourth loop region of the egg white outer membrane protein complex, the duct of *Salmonella adventitia*, and *Salmonella mycetocyte* peripheral protein-derived disulfide bond isomerase to help in disulfide arrangement. Similarly, a mixture of different antigens in safe preparations containing reasonable adjuvants and formulations that can inspire both humoral and cell-interceded reactions, ideally through mucosal rather than parenteral administration, would be an energizing development.

This patent review describes recent innovations in and current opinions on the patents published between 2010 and 2022. A formulation with novel adjuvants like Hansenula and expression vectors was found to play a critical role in the evolution of patents over the years. Clinical evidence of the enduring immune protection of these new HBsAg-based vaccines with these adjuvants makes us hopeful about future improvements in the area of HBV prevention and treatment. It should therefore be noted that the bar is high for commercial introduction of a novel standard HBV vaccine given the overall achievement of the existing promoted prophylactic vaccines that are in use. Regulatory approval of a new vaccine would need convincing evidence of the inclusion of patients that are non-responsive and hyporesponsive to the currently available HBV vaccine, long-lasting immunity with a single dose of the vaccine, and a strong oral or nasal formulation for the pediatric age group.

## Figures and Tables

**Table 1:** List of some HBV therapeutic vaccine clinical trials related to platform technology.

Study Type	Type of Vaccine	Type of HBV Antigen	Intervention	Reference
Non-randomized	Recombinant vaccines	S	Hepavax-Gene TF	[51]
Non-randomized	Recombinant vaccines	Pre-S2, S	ENGERIX-B	[52]
Non-randomized	Recombinant vaccines	S, C	HBsAg + HBcAg	[53]
Randomized	Recombinant vaccines	S, C	NASVAC (HBsAg + HBcAg)	[54]
Randomized	Immune complex vaccines	S	YIC (HBsAg-HBIG + allum adjuvant)	[55]
Randomized	Immune complex vaccines	S	YIC (HBsAg-HBIG + allum adjuvant)	[56]
Randomized	DNA vaccines	Pre-S2, S	CMV-S2.S with NUC	[57]
Randomized	DNA vaccines	Pre-S2, S	DNA pSG2.HBs and MVA vaccine (MVA.HBs) Vaccination alone or with NUC	[58]
Randomized	DNA vaccines	Pre-S2, S	ED-DNA.PS2.S with NUC	[59]
Randomized	DNA vaccines	Pre-S2, S	ED-DNA.PS2.S with NUC	[43]
Randomized	DNA vaccines	Pre-S2, S, C, P	HB-110 with NUC	[60]
Randomized	Yeast-derived vaccines	S, C, X	GS-4774 with NUC	[61]
Non-randomized	Yeast-derived vaccines	S, C, X	GS-4774 with NUC	[62]
Randomized	Yeast-derived vaccines	S, C, X	GS-4774 with NUC	[63]
Randomized	Adenoviral vectored vaccines	S, C, P	Human adenoviral type 5 vector (TG1050) with NUC	[42]

C = HBV core coding region, Core (18–25) amino acid sequence = KSSQYIKANSKFIGITEAAAFLPSDFFPSV, ELISpot = enzyme-linked immunospot assay, HBcAg = HBV core antigen, HBeAg = HBV e antigen, HBsAg = HBV surface antigen, ICS = intracellular staining assay, IFN = interferon therapy, NUC = nucleotide therapy, P = HBV polymerase coding region, Pre-S1 = HBV large surface/pre-surface-1 coding region, Pre-S2 = HBV medium surface/pre-surface-2 coding region, S = HBV small surface coding region.

**Table 2:** Granted Patents/Patent Applications for Hepatitis B Vaccines.

Patent Application Number	The Assignee of the Grant	Claim	Publication Date
CN114210310A	Wuhan Ruyi Medical Instr Co., Ltd., China	An immunoadsorption material for the hepatitis B virus and HBsAg is provided by the invention, together with a method for its production and use. A hepatitis B virus antibody solution and agarose microspheres are coupled during the coupling reaction process of aldehyde crosslinking, according to the method. To effectively lower the levels of the hepatitis B virus and HBsAg in a patient’s blood, the immunoadsorption material can directly, effectively, and selectively adsorb the hepatitis B virus and HBsAg from plasma.	22 November 2022
EP3710049A4	University of Washington	HBV PreS1 and/or PreS2, as well as S-HBsAg sections of the HBV envelope protein, are used as a therapeutic vaccine for the disease.	5 January 2022
CN113544148A	Humabs BioMed, Bellinzona, Switzerland	The antibodies and antigen-binding fragments described in the current disclosure bind to the antigenic loop region of the hepatitis B surface antigen (HBsAg) and neutralize both hepatitis B and hepatitis D virus infection (HDV). The disclosure also includes information on the epitopes that these antibodies and their antigen-binding fragments bind to, fusion proteins that contain the antigen-binding fragments, the nucleic acids that code for these antibodies and antibody fragments, and the cells that produce them.	22 October 2021
US10793866B2	Dr. LifeSciences Group Limited	This invention disclosed a method to create an edible vaccine based on an expression platform for the N-terminal yeast surface display that would protect and treat people against infection with the hepatitis B virus.	6 October 2020
US10653772B2	Tianjin Hemu Jianmin Biotechnology Co., Ltd.	A hepatitis B therapeutic vaccination based on inactivated entire recombinant Hansenula polymorpha cells producing HBsAg is being developed. The intracellular level of expression of HBsAg in the recombinant Hansenula polymorpha cells is 6–10 g HBsAg per 108 cells.	19 May 2020
CN110898219A	Institute of Biophysics, Chinese Academy of Sciences	The hepatitis B virus surface antigen peptide and ferritin are linked by a linker. The connector is flexible while the ferritin is bacterial, and the hepatitis B virus surface antigen peptide segment comprises a preS1 peptide segment.	24 March 2020
WO2019013361A1	Beacle Inc., Kagoshima University	This hepatitis B vaccine comprises formed surface antigen particles in which only hepatitis B virus L proteins or mutants of said L proteins are pooled on the lipid membrane.	17 January 2019
US10058606B2	TheVax Genetics Vaccine Co., Ltd.	In this innovative vaccine, the following components are used: (i) a hepatitis B x antigen mutant with no amino acid domain, (ii) a domain for protein transduction, and (iii) a domain that binds to either CD91 receptors or antigen-presenting cells (APCs). A linker, a translocation peptide, and a T cell sensitivity signal-transducing peptide make up the antigen transduction domain of this fusion polypeptide.	28 August 2018
CN106928372A	Application filed by Peking University Shenzhen Graduate School	This invention relates to a recombinant hepatitis B antigen which is prepared by linking the hepatitis B virus preS region, the influenza virus hemagglutinin signal peptide region, the influenza virus transmembrane region, and the intracellular region and carrying out expression. It has the potential to be transformed into a novel preventative and therapeutic hepatitis B vaccine in medical science.	7 July 2017
CN106701825A	Institute of Biophysics of Chinese Academy of Sciences	This vaccine includes a nucleic acid sequence formed through introducing s-HBsAg and the immune co-stimulator molecule LIGHT to the type-5 adenovirus vector. The vaccine has a significant therapeutic effect on hepatitis B.	24 May 2017
CN106421774A	Institute of Biophysics of Chinese Academy of Sciences	The vaccine comprises a hepatitis B virus envelope protein from the preS1 region and can effectively prevent HBV from infecting the host, and has a therapeutic effect on chronic hepatitis B virus infection; its effect is significantly better than the existing traditional HBV vaccine.	22 February 2017
CN105727280A	Tianjin Jianmin harmony Biotechnology Co., Ltd.	This is a hepatitis B therapeutic vaccine based on heat-inactivated all-recombinant Hansen USA cells capable of expression of HBsAg. The vaccine is based on the Hansenula expression system platform; by releasing IFNγ in liver cells with HBsAg-specific CTLs, it targets cells infected with HBV.	6 July 2016
CN105061591A	Second Military Medical University (SMMU)	This invention provides an anti-hepatitis B virus surface antigen fully humanized antibody, A3D5, and the gene encoding the antibody. The A3D5 antibody is capable of specifically binding to the HBsAg protein, has better HBV-neutralizing activity, and may prevent the process of infection-related hepatitis, cirrhosis, and liver cancer mediated by HBV.	18 November 2015
CN104043120A	Simcere Pharmaceutical pharmaceutical company	The immunogenic activity of hepatitis B core and surface antigens was utilized to treat HBV infection and HBV-mediated illnesses, and a method for treating HBV infection is included in the sulpho-oligodeoxynucleotide.	17 September 2014
CN111363728A	Wuhan University, China	The influenza A virus is taken as a vector, the hepatitis B virus gene is integrated into the genome of the influenza virus through reverse genetics technology, and the recombinant influenza virus can be stably passaged in a host cell or a chicken embryo.	18 June 2014
CN103566369A	Cancer Hospital and Institute, Chinese Academy of Medical Sciences, CAMS, and Peking Union Medical College, PUMC.	This invention reports a hepatitis B vaccine that induces the body to generate specific immunity under a chronic hepatitis B virus infection state. This inventive hepatitis B vaccine contains Toll-like receptor agonists as an immune enhancer.	12 February 2014
CN102949717A	China center for diseases control and prevention, National Institute for Virology, Beijing, China	This is a genetic engineering vaccine that comprises the hepatitis B virus surface antigen, an aluminum hydroxide adjuvant, and a polyinosinic acid-polycytidylic acid adjuvant. The vaccine can be used for treating chronic hepatitis B.	6 March 2013
CN102199217A	Third Military Medical University (TMMU). Chongqing, China.	This fusion protein is obtained by inserting hepatitis B virus multi-epitope fusion peptide formed by serially connecting HBsAg313-321, HBsAg335-343, Pol150-159, Pol455-463, and Padre epitopes through connecting peptide between the amino acid at the 78th position and the amino acid at the 79th position of a hepatitis B virus core. The obtained fusion protein has strong immunogenicity and therapeutic effects on hepatitis B infection.	28 September 2011
CN101942013A	Third Military Medical University (TMMU). Chongqing, China.	This innovation offers fresh approaches and techniques for the creation of a potent therapeutic vaccine against hepatitis B and has the potential to reduce immune tolerance to the disease, restore cell-mediated immune function, and effectively inhibit and eradicate the hepatitis B virus.	12 January 2011
CN101618211A	Zhuhai Lianbang Pharmaceutical Co., Ltd., China	This hepatitis B polypeptide vaccine contains polypeptides that can stimulate a CTL reaction, and has a polypeptide sequence that contains one mutational amino-acid residue with the mutation positioned at an N-terminal end or a C-terminal end of the peptide segment. The vaccine contains one or more types of polypeptides and is used for treating chronic hepatitis B infection.	6 January 2010

## Data Availability

Not applicable.
